# Effect of Sulodexide on Urinary Biomarkers of Kidney Injury in Normoalbuminuric Type 2 Diabetes: A Randomized Controlled Trial

**DOI:** 10.1155/2015/172038

**Published:** 2015-03-31

**Authors:** Bancha Satirapoj, Wisit Kaewput, Ouppatham Supasyndh, Prajej Ruangkanchanasetr

**Affiliations:** Division of Nephrology, Department of Medicine, Phramongkutklao Hospital and College of Medicine, Bangkok, Thailand

## Abstract

Glycosaminoglycans or sulodexide has shown benefits in early experimental diabetic nephropathy (DN) models, but its efficacy in patients with early stage of DN is unknown. *Methods*. Twenty patients were randomly assigned to the placebo group and another 20 patients were randomly assigned to receive sulodexide 100 mg/day for 14 weeks. Primary outcome was a change of urinary TGF-beta1, albuminuria, and glomerular filtration rate (GFR). All patients had stable metabolic profiles for at least 90 days before randomization. *Results*. Urinary TGF-beta1 increased significantly in the placebo group but did not change significantly in the sulodexide group. Additionally, the mean change of urine TGF-beta1 in the placebo group was significantly higher than that in the sulodexide group (8.44 ± 9.21 versus 2.17 ± 6.96 pg/mg Cr, *P* = 0.02). Mean changes of urinary albumin were 15.05 ± 30.09 *μ*g/mg Cr (*P* = 0.038) in the placebo group and 13.89 ± 32.25 *μ*g/mg Cr (*P* = 0.069) in the sulodexide group. No consistent patterns of side effects were observed. *Conclusion*. In this 14-week trial, benefits of sulodexide in preventing the increase of urinary TGF-beta1 were observed in patients with normoalbuminuric type 2 diabetes. The study suggests that sulodexide treatment may provide additional renoprotection in early stage DN. This trial is registered with TCTR20140806001.

## 1. Introduction

Despite advances in care over the past 20 years, diabetic nephropathy (DN) remains the single largest cause of patients with diabetes, and additional therapeutic approaches beyond glycemic and hypertensive control should be employed to reduce the rate of progression of nephropathy [1]. More innovative strategies are needed to prevent and treat DN. In fact, several clinical trial results have been disappointing [2–4].

Multiple metabolic pathways are proposed as the major mediators of DN; chronic inflammation and activation of the immune system are also involved in the pathogenesis of DN [5]. Several studies suggest that intrinsic renal cells are able to produce inflammatory cytokines and growth factors, including transforming growth factor-beta1 (TGF-beta1), having also been implicated in the progression of DN [6]. Induction of TGF-beta1 promotes the accumulation of the renal extracellular matrix [7] and inhibits TGF-beta1 action by injecting neutralizing antibodies and gene therapy demonstrates the suppression of the matrix accumulation in experimental kidney diseases [8, 9]. Previous investigators showed that the early features of diabetic renal involvement, which increased matrix mRNAs, are largely mediated by increased endogenous TGF-beta1 activity in the kidney and can be significantly attenuated by treating with neutralizing anti-TGF-beta1 antibodies [10].

Promising preclinical data suggests that glycosaminoglycans (GAGs) may prevent or attenuate DN in patients with albuminuria [11, 12]. In a study involving streptozotocin diabetic rats, daily treatment with GAG prevented structural changes in the glomerular basement membrane and changes in the albumin excretion rate, without affecting GFR [13, 14]. Proposed mechanisms of renoprotective effects of GAG include decreasing TGF-beta1 in DN. In vitro and animal model of diabetes mellitus-induced glomerulosclerosis, daily administration of GAG prevented the high glucose-mediated induction of TGF-beta1 mRNA expression, overexpression of renal TGF-beta mRNA and albuminuria [15]. It has been also shown that activity of the analog of GAG inhibits heparanase enzyme that is a key player for the development of DN and interacts with the regulation and the effects of TGF-*β* [16]. The data suggest the potential value of GAG related to an anti-TGF-beta1 effect for preventing patients with early signs of diabetic nephropathy, but this has not been established in patients. The authors, therefore, tested the effect of a short-term 14-week GAG or sulodexide treatment on urine TGF-beta1 and urine albumin in subjects with type 2 diabetes and normoalbuminuria.

## 2. Materials and Methods

### 2.1. Study Population

This was a 14-week randomized controlled study conducted among type 2 diabetes patients with normoalbuminuria at the Outpatient Medicine Clinic, Phramongkutklao Hospital, Bangkok, Thailand. The study was approved by the Institutional Review Boards of the Phramongkutklao Hospital and College of Medicine. Treatment protocol randomized patients using block randomization by computer in blocks of four. Inclusion criteria included age, 18 years or older, stable standard treatment with antiglycemic agents, and RAAS inhibitors within three months before starting the study. Exclusion criteria included type 1 diabetes, active malignancy, severe heart, lung or liver disease, stroke, chronic infection, for example, tuberculosis within one year of starting the study, and any immunological or inflammatory disorders. Signed informed consent was obtained from all subjects after a thorough discussion of the protocol, its rationale, and potential risks.

### 2.2. Intervention

Eligible patients were randomly assigned to two groups. One group ingested sulodexide composed of 80% fast-moving heparin and 20% dermatan sulfate (Vessel 2F, Alfa Wassermann SpA, Bologna, Italy), 100 mg orally daily for 14 weeks. The other group ingested a placebo with identical appearance to sulodexide, in a similar manner. Complete medical history and physical examination were performed on all subjects. Adherence was monitored by pill counts during each visit.

### 2.3. Laboratory Investigation

Complete blood counts and comprehensive serum chemistries were measured at baseline and during treatment at week 14. All subjects fasted for at least 12 hours overnight before all blood drawing. Fasting plasma glucose level was measured by glucose oxidase method [17]. Urine TGF-beta1 was analyzed by enzyme-linked immunosorbent assay (IBL-America, Inc., Minneapolis, MN). Urinary albumin and creatinine concentrations were measured, and the ratio of urinary albumin and creatinine concentrations was expressed as the urinary albumin creatinine ratio (UACR). Patients with normoalbuminuria were defined as UACR < 30 mg albumin/g creatinine in at least two of the last three urine specimens. A positive aspect of the study design was that the urine measurements at baseline and 14 weeks were performed on 3 separate urine specimens.

### 2.4. Statistical Analysis

The estimated sample size was calculated and 25 patients per treatment arm were sufficient to detect the difference of urine TGF-beta1 using a 2-sided alpha of 0.05 and 80% power between treatment groups [18]. Data are given as means ± SD for continuous variables or as a percentage in categorical variables. Normal data distribution was confirmed by the Kolmogorov-Smirnov test. Data were analyzed using Student's *t*-test and Fisher's exact test for comparisons at baseline and after treatment. Internal group changes were evaluated using paired *t*-tests. All statistical analyses were performed using SPSS version 16.0 for Windows and statistical significance was set as *P* < 0.05.

## 3. Results

### 3.1. Characteristics of Subjects

**Figure 1:**
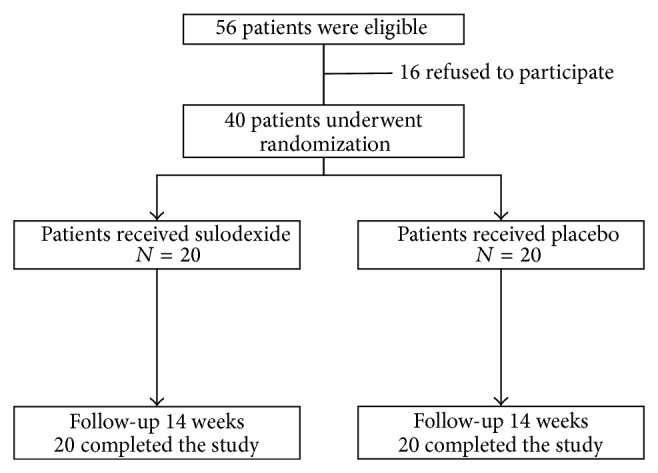
Trial flow of patients.

A total of 86 patients were screened for possible study enrollment. Fifty-six patients were eligible according to the entry criteria (Figure 1). Sixteen patients refused to participate. Thus, 40 patients actually received the treatment or placebo medications. Twenty patients were assigned to the placebo group, and 20 individuals to the sulodexide group. All patients completed the study and were 100% adherence based on pill counts to the medical prescription.

Characteristics of the study population are shown in Table 1. No significant differences were found in age, sex, body weight, duration of diabetes, retinopathy, frequency of hypertension and ACEI/ARB treatment, primary renal disease, or comorbid diseases.

### 3.2. Metabolic Outcomes after Treatment

During the whole study period, patients did not alter their usual diet, insulin, oral hypoglycemic agents, or antihypertensive treatment. Routine blood chemistry and hematologic parameters did not significantly change in both groups (data not shown). Moreover, body weight, fasting plasma glucose, hemoglobin A1C, LDL-cholesterol, systolic blood pressure, and diastolic blood pressure were comparable in both groups and did not change significantly throughout the study (Table 2).

### 3.3. Renal Outcomes after Treatment

Urinary albumin, urinary TGF-beta1, serum creatinine, and estimated GFR during the period of the study are shown in Table 3. At baseline renal parameters, no significant differences were observed in urine albumin, urine TGF-beta1, serum creatinine, and estimated GFR between the two groups. Urinary TGF-beta1 increased significantly in the placebo group (14.55 ± 10.86 to 22.99 ± 13.78 pg/mg Cr, *P* = 0.001) and this increase was significantly greater than the changes in the sulodexide group (8.44 ± 9.21 versus 2.17 ± 6.96 pg/mg Cr, *P* = 0.02). Urine albumin increased from baseline in the placebo group, by 15.05 ± 30.09 *μ*g/mg Cr (*P* = 0.038), while no significant change was found in the sulodexide group, by 13.89 ± 32.25 *μ*g/mg Cr (*P* = 0.069). However, the increase in urine albumin did not significantly differ in both groups (*P* = 0.970). In addition, no difference was found between the two groups with regard to serum creatinine or estimated GFR at baseline, during and at the end of study.

### 3.4. Safety Profile

During the 14-week study, no serious adverse events related or unrelated to sulodexide were reported in both groups. One subject developed abdominal discomfort from gastritis after taking sulodexide for four weeks. This event improved within one week after supportive treatment was given. Heartburn was also noted in two patients in the placebo group. No substantial difference was observed between groups regarding the prevalence and types of adverse event.

## 4. Discussion

The present study constitutes the first randomized, placebo-controlled trial of oral sulodexide in patients with type 2 diabetes with normoalbuminuria. The increase in urinary TGF-beta1 in the placebo group was significantly greater than in the sulodexide group. Thus, sulodexide showed the potential role of renoprotective effects on kidney injury in early stage DN. However, other renal injury biomarkers including urine albumin, serum creatinine, and estimated GFR did not significantly differ in the placebo group.

Glomeruli from humans with DN showed a striking increase in immunoreactive TGF-beta1 protein [19] and multiple reports have indicated the involvement of TGF-beta1 and growth factors in the pathogenesis of DN [20, 21]. TGF-beta1 is increased in glomeruli during the early phase after streptozotocin induction of diabetes and is a key factor involved in the pathogenesis of basement membrane thickening and extracellular matrix accumulation [22]. Our results supported that GAG or sulodexide administration showed significantly beneficial effects inhibiting rising urinary TGF-beta1 in early stage of DN. The positive effect of GAG on urine TGF-beta1 in this study was consistent with previous studies, showing that GAG, like TGF-beta1 antisense oligonucleotides, suppressed high glucose-induced TGF-beta1 levels [23]. Moreover, GAG prevented increased glomerular and tubular expression of TGF-b1 mRNA in long-term diabetic rats and hyperglycemia-induced TGF-beta1 mRNA and protein overexpression in mesangial cells [15]. Therefore, the most plausible explanation of the prevention in increased urine TGF-beta1 is a favorable effect of sulodexide on the decreased renal pathological damage and extracellular matrix formation related to TGF-beta1 activity.

Limited information regarding the effect of GAG on renal injury biomarkers in patients with diabetes and normoalbuminuria has been reported. A number of studies have confirmed the renoprotective action of sulodexide, which is a formulation composed of the two GAGs, 80% fast-moving heparin and 20% DS in patients with microalbuminuria and macroalbuminuria [24–27]. However, several reports revealed a possible neutral effect of GAG on albuminuria and renal function especially advanced stage of DN [28, 29]. The reasons for these differing results are probably the GAG preparations, different routes of administration, and type of commercially available GAG used. Additionally, sulodexide reduced proteinuria and TGF-beta1 in early kidney disease model of radiation nephropathy, but it did not affect urinary biomarkers of kidney injury in a chronic model of diabetic kidney disease [30]. These results may be explained in part by its lack of efficacy in recent clinical trials of DN.

Albuminuria is a dominant feature of developing DN and all therapeutic interventions inducing a reduction of the albumin excretion rate have a protective effect on renal function [5]. Experiments in diabetic rats demonstrated that GAGs prevented diabetes-induced albuminuria, loss of anionic sites, thickening of the GBM, and glomerulosclerosis [14]. In addition, it provided renoprotection by suppression of renal VEGF synthesis independently of glomerular basement membrane ionic permselectivity [31]. Several studies of the linkage between sulodexide and decreasing albuminuria in patients with documented DN have been mounting [24–27], but the results of our study do not support an effect of sulodexide on albuminuria and renal hemodynamics, reflected by serum creatinine and GFR. Our findings support the notion that GAG treatment was capable of reverting the established renal lesions in the diabetic rats [13]. In addition, a greater effect of sulodexide on albuminuria was observed in patients with high albuminuria than in those with low albuminuria [25, 26]. Thus, the hypoalbuminuric effect of oral sulodexide might be particularly evident in patients with significant moderately increased urine albumin, but not evident in patients with type 2 diabetes and normoalbuminuria. However, the sample size in the study was calculated from the difference of urine TGF-beta1 levels. Therefore, no significant differences in albuminuria were found between the two groups; the sample size might have been too small for statistical analysis.

Regarding the side effects of treatment, we could not find serious events in the sulodexide-treated group. However, one subject developed abdominal discomfort from gastritis after taking sulodexide for four weeks. This event improved within one week after supportive treatment. Our results demonstrated minimal serious side effects with oral doses of 100 mg sulodexide daily used over 14 weeks. Therefore, the treatment regimen could be well tolerated in patients with type 2 diabetes. However this treatment was tested only with short-term protocols, so the long-term consequences of these approaches remain unknown.

Despite the limitation in the subgroup analysis due to the small sample size, the additive anti-TGF-beta1 effect of sulodexide in patients with well-controlled BP and already receiving ACEI/ARB therapy is noteworthy because it promises a favorable effect on the dismal evolution of DN. Several limitations were associated with the present study. First, the long-term outcomes of sulodexide treatment in patients with type 2 diabetes were not demonstrated in this study. No proof has demonstrated that the decreasing quantity of urine TGF-b will have long-term effects on clinical end points. Second, no difference of urine albumin and renal function was found at the end of the study between the sulodexide and placebo groups.

In conclusion, this study provided evidence that a short course of GAG prevents rising urine TGF-beta1 levels in patients with type 2 diabetes with normoalbuminuria. Together with previous results, these data indicate the possibility that GAG therapy may represent a new therapeutic option to prevent DN. These effects may provide a rationale for early pharmacological intervention aimed at ameliorating TGF-beta1-related nephropathies in patients with type 2 diabetes.

## Figures and Tables

**Table 1:** Baseline characteristics of study participants.

Characteristics	Sulodexide	Placebo
	(*N* = 20)	(*N* = 20)
Age (years)	62.85 ± 8.04	63.7 ± 10.35
Male *n* (%)	13 (65%)	12 (60%)
Body weight (kg)	72.9 ± 13.59	66.16 ± 13.11
DM duration (years)	9.4 ± 7.6	6.15 ± 5.2
Retinopathy *n* (%)	3 (15%)	4 (20%)
ACEI/ARB *n* (%)	15 (75%)	14 (70%)
Diuretics *n* (%)	4 (20%)	4 (20%)
Comorbid diseases *n* (%)		
Coronary artery disease	1 (5%)	1 (5%)
Stroke	0 (0%)	1 (5%)
Hypertension	18 (90%)	17 (85%)
Gout	0 (0%)	1 (5%)

Data presents as mean ± SD, with analysis by *x* ^2^ or Fisher's exact tests for categorical variables and Student's *t*-test for continuous variables.

**Table 2:** Changes from baseline metabolic parameters.

Parameters	Sulodexide (*N* = 20)	Placebo (*N* = 20)	*P* value
Baseline BW (kg)	72.9 ± 13.59	66.16 ± 13.11	0.119
Change at end point (mmHg)	0.24 ± 1.04	−0.36 ± 1.44	0.144
Baseline SBP (mmHg)	128.8 ± 6.89	129 ± 12.01	0.949
Change at end point (mmHg)	−1.25 ± 10.47	−2.6 ± 15.01	0.743
Baseline DBP (mmHg)	73.85 ± 8.37	76.65 ± 8.16	0.291
Change at end point (mmHg)	−1.6 ± 9.48	−2.15 ± 8.94	0.851
Baseline FPG (mg/dL)	133.4 ± 31.29	142.38 ± 33.85	0.389
Change at end point (mg/dL)	1.65 ± 60.85	1.73 ± 42.35	0.818
Baseline HA_1_C (%)	7.41 ± 1.63	6.89 ± 0.94	0.232
Change at end point (%)	−0.1 ± 1.49	−0.05 ± 0.66	0.897
Baseline LDL (mg/dL)	86.8 ± 31.39	91.75 ± 31.77	0.623
Change at end point (mg/dL)	−4.05 ± 34.51	−2.6 ± 26.24	0.882

Data are mean SD; week 14 values compared with baseline.

**Table 3:** Changes from baseline biomarkers of kidney injury.

Parameters	Sulodexide (*N* = 20)	Placebo (*N* = 20)	*P* value
Baseline urine TGF-beta1 (pg/mg Cr)	14.85 ± 8.02	14.55 ± 10.86	0.920
Change at end point (pg/mg Cr)	2.17 ± 6.96	8.44 ± 9.21^a^	0.020
Baseline UACR (*µ*g/mg Cr)	7.44 ± 7.36	8.17 ± 8.21	0.766
Change at end point (*µ*g/mg Cr)	13.89 ± 32.25	15.05 ± 30.09^a^	0.907
Baseline serum creatinine (mg/dL)	1.00 ± 0.29	0.97 ± 0.25	0.728
Change at end point (mg/dL)	−0.01 ± 0.15	−0.06 ± 0.11	0.285
Baseline estimated GFR (mL/min/1.73 m^2^)	77.89 ± 34.21	76.17 ± 24.04	0.855
Change at end point (mL/min/1.73 m^2^)	4.2 ± 19.3	7.31 ± 14.2	0.565

Data are mean ± SD; week 14 values compared with baseline: ^a^ *P* < 0.05.
